# P‐tau217 significance as marker of brain Abeta and tau pathologies

**DOI:** 10.1002/alz.089427

**Published:** 2025-01-03

**Authors:** Nicolas R. Barthélemy, Yingxin He, Gemma Salvadó, Shorena Janelidze, Yan Li, Nupur Ghoshal, Soumya Mukherjee, Kanta Horie, Salvatore Spina, Lawren VandeVrede, Ross W. Paterson, Richard J. Perrin, Tammie L.S. Benzinger, Adam L. Boxer, Audrey Gabelle, Chihiro Sato, Suzanne E. Schindler, Eric McDade, Brian A. Gordon, Oskar Hansson, Randall J. Bateman

**Affiliations:** ^1^ Washington University School of Medicine, St. Louis, MO USA; ^2^ The Tracy Family SILQ Center, St. Louis, MO USA; ^3^ Clinical Memory Research Unit, Department of Clinical Sciences, Lund University, Lund Sweden; ^4^ University of California San Francisco, San Francisco, CA USA; ^5^ UCL Queen Square Institute of Neurology, London United Kingdom; ^6^ Washington University in St. Louis, St. Louis, MO USA; ^7^ University of California San Francisco (UCSF), San Francisco, CA USA; ^8^ Montpellier Excellence University, Montpellier France; ^9^ Washington University in St. Louis School of Medicine, St. Louis, MO USA; ^10^ Clinical Memory Research Unit, Department of Clinical Sciences Malmö, Faculty of Medicine, Lund University, Lund Sweden; ^11^ The Tracy Family SILQ Center, Washington University School of Medicine, St. Louis, MO USA

## Abstract

**Background:**

P‐tau217 has emerged as a compelling alternative to long‐established p‐tau181 to accurately measure tau modifications in biofluids in response to brain Abeta and tau deposition in Alzheimer’s disease (AD). Understanding the specificity and significance of p‐tau217 changes over AD stages is critical to interpret its potential response to treatments against Abeta and tau aggregation.

**Methods:**

We measured p‐tau217 phosphorylation by mass spectrometry. We analyzed brain, cerebrospinal fluid (CSF) and plasma samples from participants with late‐onset AD (LOAD), dominantly‐inherited AD (DIAD) and primary tauopathies.

**Results:**

Soluble and insoluble tau species extracted from LOAD and DIAD brains are p‐tau217 hyperphosphorylated [1‐2]. P‐tau217 abundance in insoluble tau extracts from AD and most primary tauopathies associates with brain tau aggregates level. In LOAD CSF and plasma, slight p‐tau217 phosphorylation changes are detected in response to early Abeta deposition in participants without cognitive symptoms [3‐4]. Early Abeta‐associated changes are observed in DIAD around 20 years before symptoms onset [5]. Subsequently, p‐tau217 increases with brain Abeta build‐up until cognitive symptoms onset. At asymptomatic stages, CSF and plasma p‐tau217/tau ratio better associates with Abeta PET than p‐tau concentration [3,6]. While brain Abeta deposition plateaus at symptoms onset, we observe p‐tau217 increases and significantly associates with Tau PET. This association weakens then disappears at dementia stage when p‐tau217 decreases. Finally, abnormal soluble p‐tau217 is observed sporadically in participants with primary tauopathies in absence of Abeta positivity [7].

**Conclusions:**

P‐tau217 is a highly accurate marker of Abeta deposition. The strong association between p‐tau217 and Abeta PET, when tau PET remains negative, supports tau hyperphosphorylation is a neuronal response to Abeta pathology independent from tau aggregation. On the other hand, p‐tau217 associates with tau‐PET at prodromal AD stage and occasionally changes in participants with primary tauopathies free of amyloid deposition. This corroborates soluble p‐tau217 also increases because of the presence of brain tau aggregates. The duality of p‐tau217 response to amyloid and tau pathology in AD complicates its utility as marker of tau aggregation. Lastly, plasma p‐tau217 use as a single marker of Abeta positivity may expose to risk of false positive AD diagnosis for patients with primary tauopathies.

